# Internet use and self-rated health among Swedish 70-year-olds: a cross-sectional study

**DOI:** 10.1186/s12877-019-1392-8

**Published:** 2019-12-23

**Authors:** Hanna Falk Erhag, Felicia Ahlner, Therese Rydberg Sterner, Ingmar Skoog, Annika Bergström

**Affiliations:** 1 Neuropsychiatric Epidemiology Unit, Department of Psychiatry and Neurochemistry, Institute of Neuroscience and Physiology, Sahlgrenska Academy, University of Gothenburg, Wallinsgatan 6, S-431 41 Molndal, Sweden; 2 Age Cap - Centre for Ageing and Health, University of Gothenburg, Wallinsgatan 6, S-431 41 Mölndal, Sweden; 3 Region Västra Götaland, Sahlgrenska University Hospital, Department of Neuropsychiatry, Gothenburg, Sweden; 4 Department of Journalism, Media and Communication, University of Gothenburg, PO Box 100, S-405 30 Gothenburg, Sweden

**Keywords:** Digital divide, Internet use, Self-rated health

## Abstract

**Background:**

The Internet is increasingly becoming an infrastructure for a number of services, both commercial, public (including health related) and personal. Using the internet have the potential to promote social interaction and social connectedness by upholding social networks and social contacts. However, Internet use is lower in older adults compared to other age groups. This digital divide is considered a risk to the health of older adults since it limits their participation in society, access and use of relevant health related information and services. This study focuses on whether there is an association between Internet use and self-rated health.

**Method:**

A cross-sectional population-based sample of 70-year-olds from The Gothenburg H70 Birth Cohort Study (*n* = 1136) was examined in 2014–16. All data was collected using structured interviews and questionnaires. Differences in proportions were tested with chi-square test and ordinary least square regression analysis was used to estimate the relationship between Internet use and self-rated health controlling for health factors, hearing and visual impairment, and social contacts.

**Results:**

There is a relationship between more frequent Internet use and good self-rated health (unstandardized *β* 0.101 *p* < 0.001), and the effect remained after adjusting for all covariates (unstandardized *β* 0.082 *p* < 0.001). Our results also show that, in comparison to health factors, Internet use is of minor importance to the SRH of older adults, since adding these improved the explanatory power of the model by approximately 400% (from 0.04 to 0.18).

**Conclusion:**

Although the direction of the relationship between more frequent interne use and better self-rated health is undetermined in the present study, it can be suggested that using the Internet informs and educates older adults, strengthening their position as active and engaged participants of society. It can also be suggested that those using the Internet report less loneliness and a possibility to establish new computer-mediated relationships within online communities. Further research needs to examine what aspects of Internet use, and in what contexts such positive perceptions arise.

## Background

Older adults constitute the segment of the population with the lowest level of Internet use (i.e. the digital divide), and are among the groups most excluded from the information society [1, 2]. For example, 58% of people over 75 use the Internet, compared to 98% of those 25–75 [3]. The Internet is increasingly becoming an infrastructure for a number of services, both commercial, public (including health related) and personal [4]. Research show that the Internet could make it possible for individuals of all ages to overcome cultural, geographic, political and physical barriers [5]. Its use among older adults have the potential to promote social interaction and social connectedness by upholding social networks and social contacts [6, 7]. It can also help reduce isolation and loneliness [4, 6], enhance social cohesion, strengthen social bonds, and develop new relationships [8–10]. Internet use also has the potential to empower older adults and contribute to their quality of life [11], facilitate their access and use of relevant health related information and services as well as their participation in society [12]. The digital divide is considered a major risk to the health and quality of life of older people, since limited access and use of the information society could diminish the individuals’ possibilities to age successfully [13].

### Social capital and connectedness in older people

Lack of social capital (i.e. the actual and potential social resources available to individuals, groups, or communities) has been associated with adverse health outcomes [14]. Social integration (i.e. the actual or perceived connectedness with others within social groups, communities and networks) and bridging social capital are closely related to psychological well-being and quality of life [15]. Research has shown an association between receiving and providing social support and improved survival rates among heart attack survivors, and decreased risk for cancer recurrence [16]. Social isolation and loneliness among older adults due to mental and or physical decline, barriers to communication, widowhood, and low income has been associated with an increased risk of mortality [9, 10, 17, and]. Feelings of loneliness, decreased social network and social support have been associated with depressive symptoms in both cross-sectional and longitudinal studies [18, 19].

### Internet use and self-rated health

Despite a large body of research pointing towards positive effects of Internet use in older adults, little attention has been paid to differences in self-rated health (SRH) among users and non-users of the Internet, and whether there is an association between Internet use and SRH. SRH is an all-inclusive, sensitive, yet non-specific measure that assesses health and predicts health outcomes in ways that are still unclear, and not necessarily identical with objective health status [20–22]. In some cases, SRH has turned out to be a better predictor of mortality than objective health indicators [23], as it integrates biological, mental, social and functional aspects of a person, including individual and cultural beliefs and health behaviors [23–25]. The use of Internet may enhance several factors deemed important to SRH of life of older adults. Research show that people who are disadvantaged in health and personal well-being are least likely to engage with Internet and digital applications [26, 27]. On the other hand, research also show that Internet use increases the self-confidence of older adults [9], and that having the ability to communicate with others through e-mail and social media despite illness, frailty, and functional disability opens up for engaging with interest [28–30]. Qualitative research show that e-leisure activities is perceived by older adults as a meaningful way to pass time that distracts the attention from a lonely situation [9], and brings joyfulness and expand social networks, which are two key factors in explaining well-being and quality of life in old age [7]. Further, digital applications can contribute to older adults continuing living independently in their own homes longer, enhancing their quality of life [12]. Older adults who use the Internet to acquire health information report significantly better general health and happiness than those who only seek information offline [31]. In addition, the effects of Internet use on depression have been found to be large and positive [6, 8]. The Internet can also be used to communicate with health care professionals, acquire information about medical issues, purchase medication and perform a variety of other tasks relevant to health [29, 30, 32, 33, and]. Although there are many health benefits of Internet use, research also show strong links between traditional social exclusion and digital exclusion, and that Internet use does not reduce isolation in vulnerable older adults, but will rather act as an additional way for those with existing social networks to stay in touch [12]. In contrast, research also show that adopting digital skills in old age can be experienced as both stressful and unpleasant, and that greater Internet use has been associated with a decline in social involvement and with increased loneliness [34]. In conclusion, research is somewhat inconsistent regarding the contribution of Internet use on SRH, and previous studies have touched upon the topic from several different perspectives. Further, there is scarce findings directly addressing the correlation between SRH and the actual use of the Internet. The aim of this study is to examine the association between Internet use and SRH among Swedish 70-year olds and to determine whether this association holds independently of health factors, hearing and visual impairment, and social contacts.

## Methods

### Sample and setting

The Gothenburg H70 Birth Cohort Studies (the H70 studies) are multidisciplinary epidemiological studies examining representative birth cohorts of older populations in Gothenburg, Sweden since 1971. In 2014–16, all men and women born 1944 on specific dates, and registered as residents in Gothenburg, were eligible for participation (*N* = 1839). Information regarding date of birth and residential addresses were obtained from the Swedish Tax Agency’s population register, which covers all persons registered as living in Sweden. Persons were considered eligible irrespective of place of living (e.g. private households, sheltered living). A total of 1203 (response rate 72.2%; 559 men and 644 women; mean age 70.5 years) agreed to participate in the study. The study comprised a one-day basic examination, and thereafter a number of additional examinations. The complete study protocol and sample has been described in detail previously [35]. In this paper, participants for whom SRH could not be established due to missing data (*n* = 39) and participants with dementia were excluded from our analysis (*n* = 28), leaving 1136 individuals.

### Self-rated health

Study participants were asked to answer the question; *“How would you rate your overall health?”* Response options ranged from “excellent”, “very good”, “good”, “moderate”, to “poor”.

### Internet use

The frequency of Internet use was measured on a 7-point scale with response options ranging from “never”, “less than once a month”, “at least once a month”, “at least once a week”, at least 4 times a week”, “daily” or “several times a day”. A dichotomized (yes/no) follow-up question captured involvement in different Internet areas, such as instant messaging, e-mail, and text messaging, communication-oriented Internet sites such as blogs and social networking, web browsing and online information search, e-shopping, online banking, reading news and blogs, watching movies and TV-series.

### Social contacts

Social contacts were defined as having contact with children, grandchildren, siblings and friends. These four categories were measured separately on a 6-point scale with response options ranging from “daily”, “at least once a week”, “at least once a month”, “at least once every 3 months”, “at least once every year” to “never”. In order to create a composite score that reflected the total amount of social contacts, all four categories were merged by their maximum scale point, meaning that a “daily” answer in at least one of the categories was coded as “daily” in the total social contacts variable (i.e. the maximum value of the total social contacts variable was 6). Feelings of loneliness were assessed using the question; *“Do you feel lonely”* with response options ranging from “never or rarely”, “sometimes” to “often”.

### Health factors

Health conditions were self-reported and ascertained by a positive answer to the question *“Have you ever been told by a doctor that you have…?”* In the present study, we used the following health conditions in our analysis; pulmonary disease (including asthma, chronic obstructive pulmonary disease, chronic bronchitis, and emphysema), cardiovascular disease (including heart attack, chronic heart failure, angina pectoris, and intermittent claudication), diabetes, stroke, depression, and anxiety. COPD was diagnosed in those who responded “yes” to the questions “do you usually cough up phlegm from your chest first thing in the morning?” and “for how many months of the year does this usually happen?” was 3 months or more. Diagnoses for psychiatric disorders (e.g. depression and anxiety) followed *the Diagnostic and Statistical Manual of Mental Disorders*, DSM-IV, DSM-IV-TR, and DSM-5 criteria as closely as possible [36]. For the purpose of this paper, the term any depression (yes/no) was used to denote those fulfilling criteria for either major or minor depression [37].

### Functional impairment

Hearing and vision impairment was assessed using the questions “do you have trouble hearing the television or doorbell even with hearing aids” and “do you have trouble reading even with glasses or contact lenses” with response options ranging from “no impairment”, “mild impairment” to “moderate and severe impairment”. For the purpose of this paper, participants with mild to severe impairment was regarded as impaired (yes/no).

### Statistical analysis

Differences in proportions were tested with Pearson’s Chi-square. Linear regression (Ordinary Least Square, OLS) was used to estimate the relationship between Internet use (independent variable or predictor) and SRH (dependent variable). Three models were used with a step-wise adjustment for all covariates (model 2: health factors and functional impairment, model 3: social contact and perceived loneliness) so that each independent variable could be assessed in terms of what it added to the relationship between Internet-use and SRH. All analyses were performed using IBM SPSS Statistics version 25, and the level of significance was set to *p* < 0.05 (two-sided).

## Results

Descriptive statistics of the sample are presented in Table 1. In total, 1136 70-year-olds participated in the study, and 69.3% of the men and 63.3% of the women used Internet daily. The online activity mentioned by most respondents was web browsing and online information search (83.6% of the men and 83.4% of the women), followed by e-mail (79.9% of the men and 79.2% of the women), online banking (75.9% of the men and 69.9% of the women), and reading news and blogs (70.2% of the men and 61.2% of the women). Communication-oriented Internet sites and social networking such as Facebook and Instagram was used by 35.4% of the men and 44.3% of the women.

**Table 1:** The characteristics of the sample divided by gender

	Male *n* = 523 (%)	Female *n* = 613 (%)	*P*-value
Self-rated health (SRH)			
*Excellent*	70 (13.4)	66 (10.8)	ns
*Very good*	190 (36.6)	221 (36.1)	ns
*Good*	190 (36.6)	214 (34.9)	ns
*Moderate*	64 (12.2)	99 (16.2)	ns
*Poor*	9 (1.7)	13 (2.1)	ns
Pulmonary disease^a^	113 (20.3)	150 (23.5)	ns
Cardiovascular disease^b^	89 (15.9)	76 (11.8)	0.044
Diabetes	68 (12.2)	56 (8.8)	ns
Stroke	35 (6.5)	42 (6.7)	ns
Depression	37 (6.7)	70 (11.0)	0.011
Anxiety	16 (2.9)	56 (8.8)	0.000
Vision impairment *(mild to severe)*	297 (54.0)	312 (49.1)	ns
Hearing impairment *(mild to severe)*	207 (37.7)	165 (25.9)	0.000
Social contacts index			
*Daily*	203 (39.8)	316 (52.1)	ns
*Once a week*	269 (52.7)	269 (45.2)	ns
*Once a month*	34 (6.7)	7 (1.2)	ns
*More seldom or never*	4 (0.8)	3 (0.5)	ns
Perceived loneliness			
*Never*	495 (89.7)	494 (77.7)	0.000
*Sometimes*	43 (7.8)	106 (16.7)	0.000
*Often*	14 (2.5)	36 (5.7)	0.000

^a^Pulmonary disease included asthma, chronic obstructive pulmonary disease, chronic bronchitis, and emphysema. ^b^Cardiovascular disease included heart attack, chronic heart failure, angina pectoris, and intermittent claudication

### Prevalence of SRH

49.7% of the men and 46.8% of the women assessed their health as excellent or very good. When combining the response options “good”, “excellent” and “very good”, the majority of both men and women (86% of men and 81.7% of the women) fell into that category

### Prevalence of health factors

The most prevalent health condition among the respondents were pulmonary disease (including asthma, chronic obstructive pulmonary disease, chronic bronchitis, and emphysema) (20.3% in men and 23.5% in women), followed by cardiovascular disease (including heart attack, chronic heart failure, angina pectoris, and intermittent claudication) (15.9% in men and 11.8% in women), diabetes (12.2% in men and 8.8% in women), and depression (6.7% in men and 11.0% in women).

### Prevalence of functional impairment

About half of the participants reported to have vision impairment (54.0% in men and 49.1% in women), and about one third of the participants reported to have hearing impairment (54.0% in men and 49.1% in women).

### Prevalence of social contacts and perceived loneliness

According to the total social contacts index, 39.8% of the men and 53.1% of the women had daily contact with children, grandchildren, siblings or friends. Among the participants, 89.9% of the men and 77.7% of the women reported that they never or rarely felt lonely.

### Association between internet use and SRH

The OLS regression models are presented in Table 2. In the unadjusted model (Model 1), we found a significant correlation between good SRH and frequent Internet use (unstandardized *β* 0.101 *p* < 0.001). For every unit increase in Internet use, the SRH increased with 0.101 units. When adjusting for all six health factors (i.e. pulmonary disease, cardiovascular disease, diabetes, stroke, depression, and anxiety), hearing, and vision impairment, the significant correlations between good SRH and frequent Internet use remained, although somewhat reduced from 0.101 to 0.089 (Model 2), and the explanatory power of the model increased from 0.041 to 0.176. All health factors included in the second model affected SRH negatively. The variables measuring hearing and vision impairment did not affect SRH when Internet use and health factors were taken into consideration. In the last regression model (Model 3), the social dimension, measured by the degree of social contact and perceived loneliness, was added. This slightly strengthened the explanatory power of the model (0.188), and the significant correlation between good SRH and frequent Internet use remained. For every unit increase in Internet use, the SRH increased with 0.082 units. Both variables on the social dimension had single significant impact on SRH with a stronger correlation for perceived loneliness than for social contacts. The impact of the health factors decreased somewhat, but differences compared to Model 2 were very small.

**Table 2:** Impact of Internet use, health factors, hearing and vision impairment, and social contacts on SRH (Ordinary Least Square, unstandardized Beta)

	Model 1		Model 2		Model 3	
Internet use	0.101	***	0.089	***	0.082	***
Pulmonary disease			−0.289	***	−0.268	***
Cardiovascular disease			−0.189	*	−0.202	*
Diabetes			−0.424	***	−0.418	***
Stroke			−0.592	***	−0.570	***
Depression			−0.675	***	−0.576	***
Anxiety			−0.315	*	−0.256	*
Vision impairment			−0.095		−0.093	
Hearing impairment			−0.007		0.005	
Social contacts index					0.095	*
Perceived loneliness					−0.250	***
Adj R2	0.041		0.176		0.188	

* *p* < 0.05 ** *p* < 0.01 *** *p* < 0.001

## Discussion

The aim of this study was to examine the association between Internet use and SRH among Swedish 70-year olds and determine whether this association held independently of health factors, hearing and visual impairment, and social contacts. Our findings indicate that there is a significant relationship between more frequent Internet use and better SRH (Model 1), and that this effect remains when the covariates (i.e. health factors, hearing and visual impairment, and social contacts) are entered into the model (Models 2 and 3). Overall, these results also show that Internet use is of minor importance to the SRH of older adults, and that health factors play a more central role, as adding these improved the explanatory power of the model by approximately 400% (from 0.04 to 0.18). All included health factors affect SRH negatively, which is in line with previous research [13]. The variables measuring hearing and vision impairment did not affect SRH when Internet use and health factors were taken into consideration. Social contacts and perceived loneliness had single significant impact on SRH with a stronger correlation for perceived loneliness than for social contacts. Contrary to Gracia & Herrero [13], who found no evidence of a relationship between Internet use and SRH, our results suggests that there is minor evidence supporting the idea that use of Internet has a relationship with SRH in older adults. However, based on our findings it also becomes evident that SRH is a complex concept, which depends on several contextual factors. Less than 20% of the participants’ SRH could be explained by the numerous factors included in this study. Research examining the sociodemographic correlates of Internet use among older adults suggests that the average user is younger and has more education and income than the average non-user [38, 39]. In our study, all participants were the same age (i.e. 70 years) with high educational attainment (i.e. 82.9% had more than primary education > 9 years and 28.5% hade university degree). The initial analyses also included sex and educational level. However, none of these factors added any explanatory power to the relationship between Internet use and SRH. Research show that the effects of gender and income on Internet use is less robust in older age groups [40]. Instead, attitudinal variables, such as self-efficacy and interest have a more vital role [39]. However, it can be hypothesized that self-efficacy (and interest) also affects SRH of the individual, as well as the fact that older adults are a heterogeneous group, which may implicate that their use of the Internet is also diverse. For instance, older adults can use the Internet for different activities, and this usage can be of different influence on benefits. It has been suggested that access and participation in the information society will promote positive health outcomes [11, 41]. The analysis of Internet use in a large and representative sample of 70-year-olds in relation to SRH is a strength of this study. Our results show that 69.3% of the men and 63.3% of the women used the Internet daily. For example, a recent national survey in Sweden estimated that 56% of adults aged 76 years and older are Internet users [3] compared with less than 10% in 2003 [42]. However, it is not clear to what extent such growth is due to the Internet revolution and the increased use of the Internet in all age-groups over time (i.e., period effects) or the movement of cohorts with higher use into older ages (i.e., cohort effects). The most common online activity was web browsing and online information search (83.6% of the men and 83.4% of the in women), followed by e-mail (79.9% in men and 79.2% in women), online banking (75.9% in men and 69.9% in women), and reading news and blogs (70.2% in men and 61.2% in women). Communication-oriented Internet sites and social networking such as Facebook and Instagram was used by 35.4% of the men and 44.3% of the women. In future epidemiological studies on SRH and Internet use in older adults, more detailed questions about the use of specific online activities in everyday life could reveal information about habits in different Internet areas. In addition, it would be valuable to include questions pertaining to what dimensions of Internet use that could improve or impair the SRH of older adults. According to the total social contacts index, 39.8% of the men and 53.1% of the women had daily contact with children, grandchildren, siblings or friends, and 89.9% of the men and 77.7% of the women reported that they never or rarely felt lonely. A previous study from the H70 studies showed that social contacts with others were related to depression in 70-year-olds born in 1930 and examined in the 1970s, but not in those born in 1901–02 and examined in the 2000s, which might reflect period changes in the ways of socializing, communicating and entertaining due to technological development [43]. Dickinson and Gregor [11] found that Internet use does not reduce isolation in vulnerable older adults, but rather act as an additional way for those with existing social networks to stay in touch. Since the late 1980s, the concept of successful aging has set the frame for discourse about contemporary aging research [44, 45]. Despite an increasing focus on the improvement of quality of life throughout the life course, there is no generally accepted definition of what it means to age actively, healthy and successfully [46]. Traditional conceptualization of successful aging refers to the bio-medical model focusing on physical health, functional and cognitive capacity [47, 48]. In relation to successful aging, it might be suggested that older adults using the Internet report less loneliness due to more frequent contact with family, friends, and the possibility to establish new computer-mediated relationships within online communities and chat rooms. It might also be suggested that frequent Internet use informs and educates older adults that strengthens their position as active and engaged participants of society.

### Limitations

Firstly, this study was cross-sectional, making causal inference between Internet use and SRH difficult. We cannot know if our more frequent Internet users had better SRH than non-users, or if their SRH positively influenced their Internet use. Using a longitudinal design would have made it possible to detect developments or changes in SRH and Internet use at both group and individual level. Secondly, due to lack of data from earlier cohorts and older age groups, data from one birth cohort of 70-year-olds was used. Including several cohorts of older adults of different ages would have strengthened the study findings since it would have made it possible to detect both time- and cohort effects. Thirdly, we did not have information about the frequency of specific online activities or what specific dimensions of Internet use that could improve or impair the SRH of older adults, which would have strengthened our findings.

## Conclusion

We found that there is an association between more frequent Internet use and better SRH although the direction of this relationship is undetermined in the present study. Further research needs to examine what aspects of Internet use, and in what contexts such positive perceptions arise, in the older population. Previous research has concluded that a) socio-economic factors are strong predictors of digital use or disengagement ([26, 27, 49], and b) persons with low socio-economic status in general have poorer SRH than persons with higher socio-economic status [50]. Socio-economic factors thus work in the same direction for the two phenomena described in this paper. Although the positive effects of Internet use on SRH was small in our study, good SRH has strong implications for life satisfaction. Therefore, the civic society should support Internet use among older people. This should be done both in terms of how previous real life activities could be transferred to online activities in order to maintain social contacts, and from the perspective of how feasible digital applications could attract new areas of activities and services for older people, which could make them feel healthier and affect their possibilities to live an independent life.

## Abbreviation

SRH: Self-rated health
